# Supplementary figures and images for: A new method for the reproducible development of aptamers (Neomers)
Source: PLoS One. 2025 Feb 12;20(2):e0311497. doi: 10.1371/journal.pone.0311497 (PMC11819540; doi:10.1371/journal.pone.0311497)

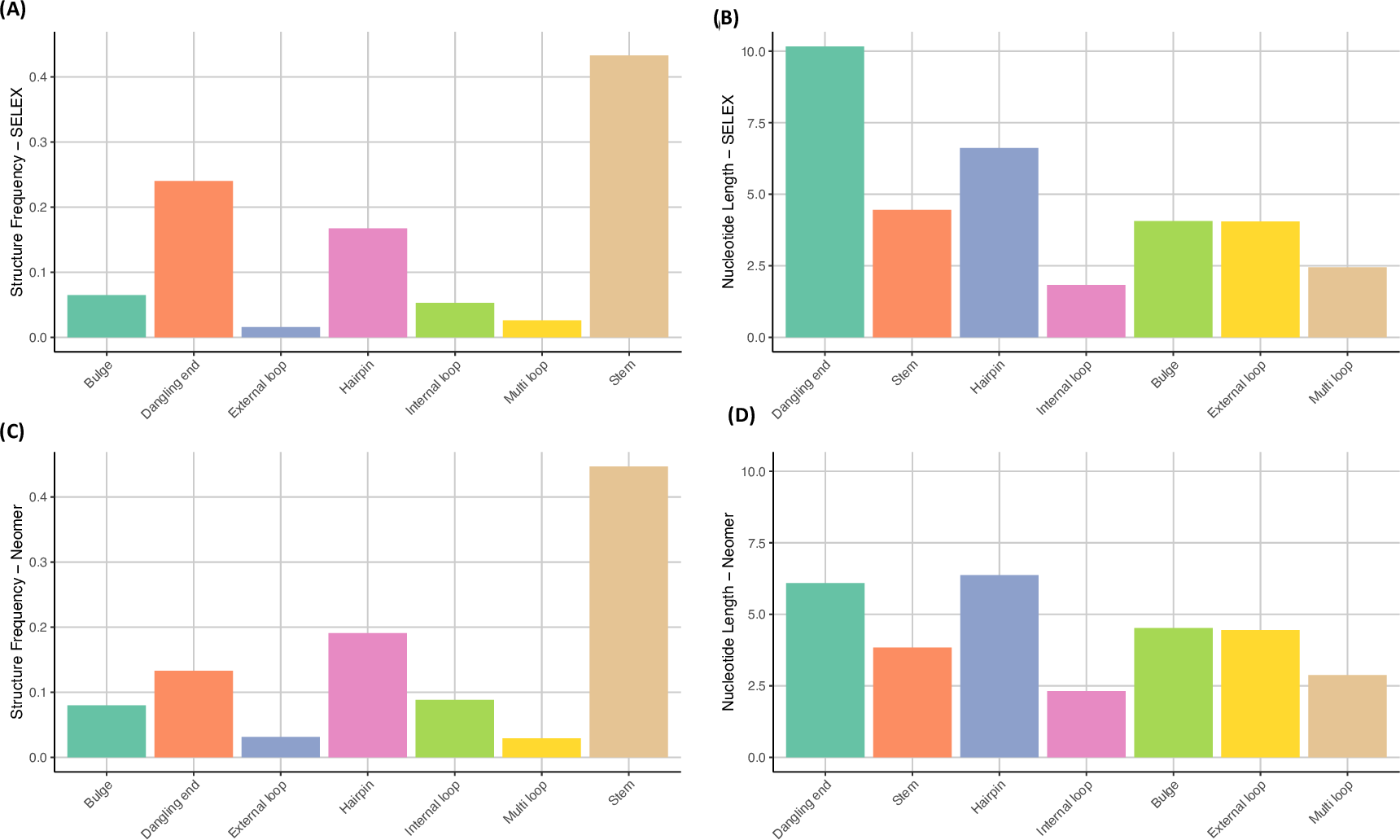

Supplement: S1 Fig — A) Average secondary structure motif frequency in each sequence across 1000 randomly generated SELEX sequences. B) Average contiguous nucleotide length of secondary structure motifs in each sequence across 1000 randomly generated SELEX sequences. C) Average secondary structure motif frequency in each sequence across 1000 randomly generated Neomer sequences. D) Average contiguous nucleotide length of secondary structure motifs in each sequence across 1000 randomly generated Neomer sequences. (TIF) [file pone.0311497.s001.tif]

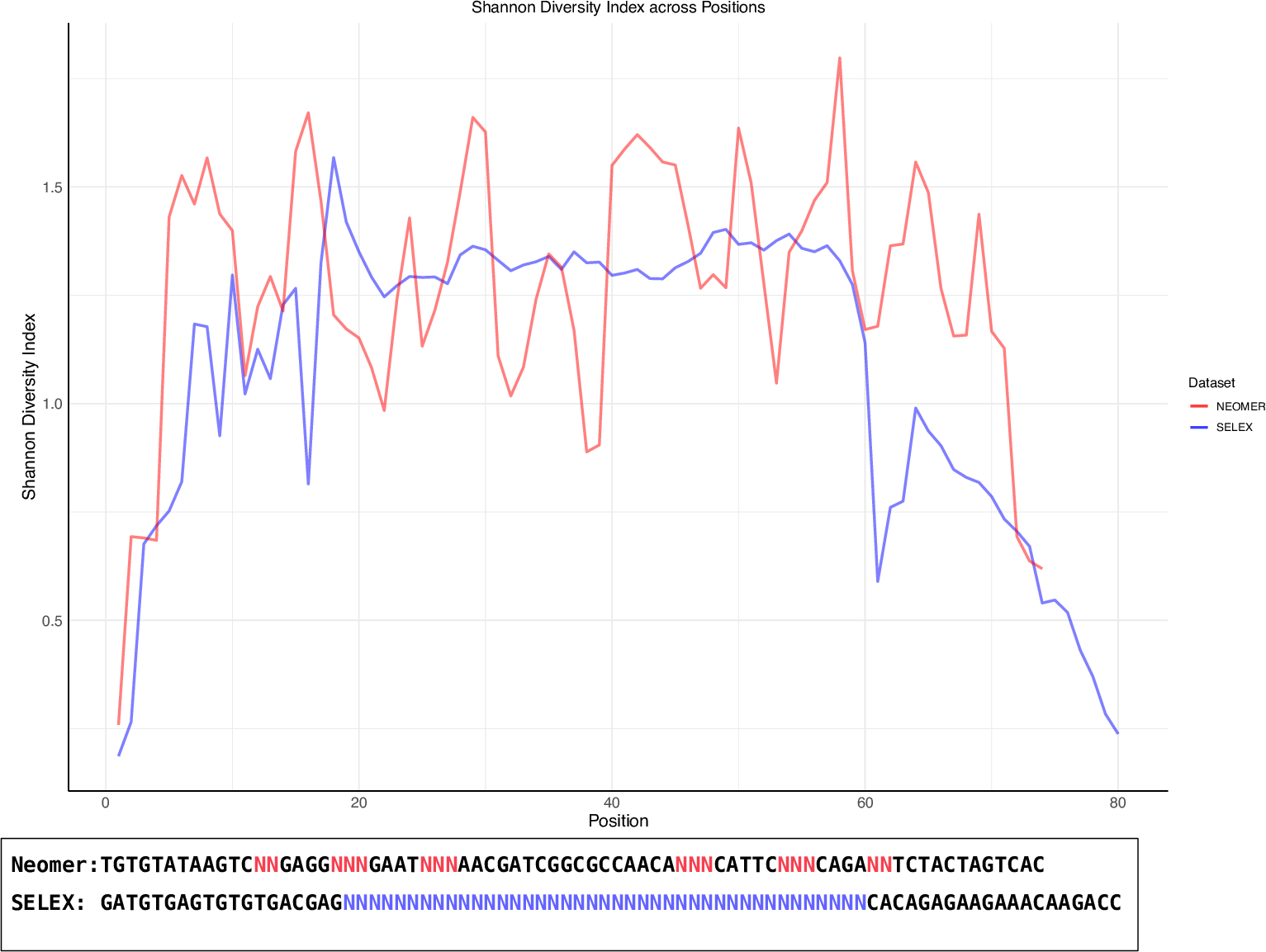

Supplement: S2 Fig — The template per position is plotted out below the plot. (TIF) [file pone.0311497.s002.tif]
