# Supplementary material for: A new method for the reproducible development of aptamers (Neomers)
Source: PLoS One. 2025 Feb 12;20(2):e0311497. doi: 10.1371/journal.pone.0311497 (PMC11819540; doi:10.1371/journal.pone.0311497)
Supplement: S1 Table — (DOCX) [file pone.0311497.s003.docx]

| **Sample Name** |  | **Module A Counts** |  | **Module B Counts** |  | **Total A + B Counts** |
| --- | --- | --- | --- | --- | --- | --- |
| IL 6 |  | 15,636,440 |  | 18,418,205 |  | 34,054,645 |
|  |  | 18,307,668 |  | 16,278,830 |  | 34,586,498 |
|  |  | 26,170,063 |  | 15,742,736 |  | 41,912,799 |
|  |  |  |  |  |  |  |
|  | Subtotal A: | 60,114,171 | Subtotal B: | 50,439,771 |  |  |
| HSA |  | 8,024,915 |  | 7,409,372 |  | 15,434,287 |
|  |  | 10,732,965 |  | 6,606,378 |  | 17,339,343 |
|  |  | 7,396,384 |  | 7,020,238 |  | 14,416,622 |
|  |  |  |  |  |  |  |
|  | Subtotal A: | 26,154,264 | Subtotal B: | 21,035,988 |  |  |
| Naive |  | 52,978,828 |  | 11,010,993 |  | 63,989,821 |
|  |  | 6,797,916 |  | 14,361,657 |  | 21,159,573 |
|  |  | 8,846,060 |  | 19,955,954 |  | 28,802,014 |
|  |  |  |  |  |  |  |
|  | Subtotal A: | 68,622,804 | Subtotal B: | 45,328,604 |  |  |
| Nickel Resin |  | 24,152,760 |  | 21,664,127 |  | 45,816,887 |
|  |  | 28,449,715 |  | 26,463,258 |  | 54,912,973 |
|  |  | 26,912,596 |  | 13,828,893 |  | 40,741,489 |
|  |  |  |  |  |  |  |
|  | Subtotal A: | 79,515,071 | Subtotal B: | 61,956,278 |  |  |
| UltraLink |  | 13,246,353 |  | 19,423,621 |  | 32,669,974 |
|  |  | 18,885,609 |  | 22,942,733 |  | 41,828,342 |
|  |  | 48,388,049 |  | 47,107,832 |  | 95,495,881 |
|  |  |  |  |  |  |  |
|  | Subtotal A: | 80,520,011 | Subtotal B: | 89,474,186 |  |  |
|  | **Total A counts:** | 314,926,321 | **Total B counts:** | 268,234,827 | **Grand total:** | 583,161,148 |
|  |  |  |  |  |  |  |
