# Supplementary material for: A new method for the reproducible development of aptamers (Neomers)
Source: PLoS One. 2025 Feb 12;20(2):e0311497. doi: 10.1371/journal.pone.0311497 (PMC11819540; doi:10.1371/journal.pone.0311497)
Supplement: S2 Table — (DOCX) [file pone.0311497.s004.docx]

| Neoer III Fwd | CAAATACGTATGAGGTCGCTCGTTCTGTGTATAAGTC |
| --- | --- |
| Neoer III Rvs | TAATACGACTCACTATAGGGATAATGTGACTAGTAGA |
| RNm NGS1-A Fwd | CCCTACACGACGCTCTTCCGATCTNNNNNNCAAATACGTATGAGGTCGCTCGTTC* |
| RNm NGS1-A Rvs | GGTCAGACGTGTGCTCTTCCGATCGGGGCGCCGATGGTT |
| RNm NGS1-B Fwd | CCCTACACGACGCTCTTCCGATCTATCACGGCGCCAACA |
| RNm NGS1-B Rvs | GGTCAGACGTGTGCTCTTCCGATCGGGTAATACGACTCACTATAGGGATAATGCTGTCTACTG |
| NGS2 Fwd | AAT GAT ACG GCG ACC ACC GAG ATC TAC ACT CTT TCC CTA CAC GAC GCT CTT CCG |
| NGS2 Rvs | CAA GCA GAA GAC GGC ATA CGA GAT GTG ACT GGA GTT CAG ACG TGT GCT CTT CC |

*Where N represents the position of the Hex Code used for NGS data analysis
