# Supplementary material for: A new method for the reproducible development of aptamers (Neomers)
Source: PLoS One. 2025 Feb 12;20(2):e0311497. doi: 10.1371/journal.pone.0311497 (PMC11819540; doi:10.1371/journal.pone.0311497)
Supplement: Attachment — Submitted filename: Rebuttal letter 11.9.24.docx [file pone.0311497.s007.docx]

Dear Editor and Reviewer #2,

We thank you for your detailed feedback regarding the Neomer library design methodology. We recognize that our initial manuscript lacked sufficient detail on the neomer design . In response to your comments, we have made substantial additions to the manuscript, particularly in the "Library Design" section of the Materials and Methods. These changes aim to provide a more comprehensive explanation of our design process and rationale.

Specifically, we have added the following information about our systematic design methodology:

1. Primer recognition sequences were designed considering 40-60% G/C content and ensuring non-hybridization.
2. A restriction site was incorporated in the middle of the library for module separation. The surrounding fixed region was designed for dual functionality, serving as both a restriction site and a primer recognition site post-restriction.
3. Internal sequences for primer recognition were designed following standard primer rules.
4. Random nucleotides were arranged in a symmetrical manner (2:3:3) with consistent fixed sequence nucleotide spacing between such regions. This approach of using blocks of random sequences (2 or 3 nucleotides) instead of single nucleotides was selected to drive higher levels of structural diversity, as hybridisation is more likely to occur contiguously for larger secondary structures.
5. Fixed sequences were designed using a random identity generator, with nucleotide substitutions made as necessary to avoid internal hybridization and keep minimum free energy (MFE) sufficiently high, preventing energy barriers to secondary structure formation.

We have also clarified our use of Vienna RNAfold to predict and minimize self-hybridization in fixed regions by selecting templates without random nucleotide identity.

These additions provide a much more comprehensive methodology for Neomer library design. We appreciate the comments, which have significantly improved the clarity and scientific rigor of our work. We believe these changes will provide a solid foundation for other researchers to understand our Neomer library design approach.

Cathal Meehan, Ph.D.

Senior Bioinformatician

Neoventures Biotechnology Europe SAS
