# Supplementary material for: A new method for the reproducible development of aptamers (Neomers)
Source: PLoS One. 2025 Feb 12;20(2):e0311497. doi: 10.1371/journal.pone.0311497 (PMC11819540; doi:10.1371/journal.pone.0311497)
Supplement: Attachment — Submitted filename: Rebuttal letter.docx [file pone.0311497.s006.docx]

Dear Editor

Thank you for the opportunity to revise our manuscript "A new method for the reproducible development of aptamers (Neomers)".

Regarding the suggestion to compare our Neomer method with traditional SELEX:

We appreciate this suggestion but assert that such a comparison would not be meaningful due to fundamental differences between the methods:

1. Fundamental incomparability: SELEX and Neomers are designed with fundamentally different principles and goals. SELEX aims to evolve high-affinity binders from a vast, diverse sequence space through multiple rounds of selection and amplification. Our Neomer approach uses a defined, reproducible sequence set to enable single-round selection and comprehensive specificity screening. These core differences in design and purpose make direct comparisons problematic and potentially misleading.
2. Reproducibility: A key advantage of our Neomer approach is its reproducibility, which SELEX fundamentally lacks. Each SELEX selection necessarily starts with a different subset of sequences from its vast possible sequence space (typically 10^15 out of 10^24 possible sequences for a 40-nt random region). This means it's impossible to replicate the starting conditions of a SELEX experiment, making each selection inherently unique. Our Neomer library, with its closed set of 4.29 x 10^9 sequences, enables truly reproducible selections - a feature that cannot be directly compared with SELEX.
3. Specificity screening capabilities: Our Neomer method allows for in silico screening against multiple targets simultaneously. This comprehensive specificity screening is not possible with traditional SELEX due to both issues raised above and represents a key advantage of our approach that cannot be directly compared. The ability to perform this screening is intrinsically linked to our use of a defined, limited sequence space - a feature that is incompatible with traditional SELEX principles.
4. 16-nt SELEX suggestion: While using a 16-nt random region in SELEX might seem to create a more comparable system, this approach would fundamentally undermine the principles and strengths of SELEX:
   - Severely limited structural diversity: A 16-nt contiguous random region would create an incredibly small amount of structural diversity due to the nucleotide hybridization problem. In a contiguous random region, nucleotides within 3 positions of each other cannot hybridize, severely limiting potential secondary structures. Our Neomer design overcomes this by interspersing fixed and random regions.
   - Loss of SELEX advantages: Reducing SELEX to a 16-nt random region would negate the key advantages of SELEX, such as its ability to sample a vast sequence space. It would no longer represent SELEX as it's understood and practiced in the field.
   - Incompatible with SELEX principles: SELEX is designed to evolve binders from a large, diverse pool. Constraining it to a small random region fundamentally alters its nature and purpose, making it neither true SELEX nor comparable to our Neomer approach.

While multiple IL aptamers have been developed using SELEX, very few have specificity data, and none have been commercialized. This highlights a critical gap in the field that our Neomer approach aims to address. We’ve rewritten sections in the introduction and discussion to further highlight this. Our method enables reproducible and specific aptamer selections at a comparable level of sensitivity to what's reported in the literature, while also providing specificity data.

We understand the reviewers' interest in comparing our method to SELEX. However, we recently received feedback on another manuscript from PlosOne “A reproducible approach for the use of aptamer libraries for the identification of Aptamarkers for brain amyloid deposition based on plasma analysis.“ suggesting that this comparison made the publication overly confusing. This comparison might have made more sense in the context of aptamarkers as we showed that 24 SELEX derived aptamarkers were outperformed by 8 Neomer derived aptamarkers. However, the final version that has now been formally accepted excludes this data. For all the reasons outlined above, the intention of this publication wouldn’t allow for SELEX and Neomer to be meaningfully compared here by desiging and performing experiments with 16nt SELEX libraries. We have included references to the literature for interleukin aptamers that stresses the issues of commercialisation and specificity in aptamer development using SELEX methods. Respectfully, we feel this adequately addresses this point.

Reviewer #1:

1. Neomer design rationale: We've expanded our explanation of the Neomer design in both the introduction and discussion. Our choice of 16 random nucleotides strikes a balance between structural diversity and statistical robustness. We acknowledge this may not be optimal for all applications and outline our plans for future meta-analysis to further optimize the design.
2. Selection rounds: We've clarified that only one round of selection was performed, addressing any confusion in the methods section.
3. Conformational changes and applications: We appreciate the reviewer's comment, but would like to clarify that conformational changes are not a primary consideration in our Neomer approach. Our focus is on identifying aptamers with the highest affinity and specificity possible for the target, which is crucial for overcoming the main hurdle in aptamer commercialization, particularly in diagnostics: achieving sufficient specificity against abundant proteins like serum albumin. Conformational changes, while important in some applications, can potentially limit the pool of high-affinity, highly specific aptamers. Our approach prioritizes these key attributes first, as no aptamers have yet received FDA or CE Mark approval for diagnostic applications, primarily due to specificity issues. We believe that functionality requiring conformational changes can be engineered into high-performing aptamers post-selection if needed, rather than constraining the initial selection process. By focusing on affinity and specificity in our selection process, we aim to address the critical barriers to commercial application of aptamers.
4. Comparison with traditional SELEX: We appreciate this suggestion but must assert that a direct experimental comparison would not be scientifically appropriate due to fundamental differences in the methods, including:
   - Lack of reproducibility in SELEX
   - Different starting conditions and library compositions
   - Single vs. multiple selection rounds
   - Ability to perform in silico specificity screening
5. Minor issues: We've addressed the issues at lines 224 and 194.

Reviewer #2:

1. SELEX library design claim: We've moderated our statement about 40 random nucleotides in SELEX and provided more context on the variability in SELEX library designs.
2. Comparison with same number of random sequences: We explain why this comparison wouldn't be meaningful due to the fundamental differences in library design and selection process between Neomers and SELEX.
3. Rationale for 16 random nucleotides: We've expanded our explanation for choosing 16 random nucleotides and clarified that this isn't necessarily the optimal number for all applications.

Reviewer #3:

1. Criteria for 16 randomized nucleotides: We've elaborated on the design criteria in both the introduction and methods sections.
2. HSA conjugation method: We've provided more details on the HSA conjugation and blocking process.
3. Flowthrough column details: This already has as much information as we could possibly add.
4. Selection rounds: We've clarified that only one round of selection was performed for both IL-6 and HSA.
5. Secondary structure statement: We've revised this section to better align with Figure 1A and clarified our meaning.
6. Figure 1B clarification: We've improved the figure legend to clarify the difference between target and naïve samples.
7. SELEX library comparison procedure: We've added more details on how this comparison was conducted.
8. SELEX library details: We've included the missing information about the SELEX library used for comparison.
9. Missing reference: We've added the missing reference for the SELEX template.
10. Single round selection justification: We've elaborated on why a single round of selection is sufficient in our approach.
11. NGS run limitations: We've explained why a single NGS run can't determine the frequency of all 4.29 x 10^9 sequences and clarified the distinction between frequency and copy number.
12. Z-score calculations: We've simplified the explanation of z-score calculations to make them more accessible.

We look forward to your response and can address any further questions or concerns.

Sincerely,

Dr. Gregory Penner
